# Exploring the Viability and Acceptance of Nudge in Public Policies for Health Promotion

**DOI:** 10.3390/healthcare12040476

**Published:** 2024-02-15

**Authors:** Teresa Forte, Gonçalo Santinha, Marta Patrão

**Affiliations:** 1 Center for Research in Neuropsychology and Cognitive and Behavioral Intervention (CINEICC), University of Coimbra, 3000-115 Coimbra, Portugal; teresa.forte@fpce.uc.pt; 2 Research Unit on Governance, Competitiveness and Public Policies (GOVCOPP), Department of Social, Political and Territorial Sciences, University of Aveiro, 3810-193 Aveiro, Portugal; marta.patrao@ua.pt

**Keywords:** health, public health, nudging, governance strategies, political science, public administration

## Abstract

Background: Behaviorally informed interventions, such as nudging, encourage actions intended to promote longer and healthier lives. Holding significant potential for influencing health policies and healthcare practices, these interventions are partaking of a shift in governance and public health policymaking. However, a substantial knowledge gap remains regarding the feasibility and appropriateness of implementing policies that draw on nudge. Methods: Ae survey on nudge’s acceptability) was adapted to the Portuguese context to access attitudes towards 16 nudge measures. The research focused on evaluating attitudes among political science and public administration BSc and MSc students from nine Portuguese universities, and analyzing the relationship between these attitudes, sociodemographic characteristics, and sociopolitical attitudes. The data analysis involved the application of descriptive and inferential statistics. Results: The participants exhibited a moderate-to-high level of approval for various nudge measures, particularly those related to nutrition and public education/awareness raising. The study identified a nuanced relationship between the level of intrusiveness of nudges and their public approval, indicating that interventions preserving the architecture of choice gathered higher acceptance compared to more intrusive approaches. Notably, approval was associated with a high level of trust in social groups and a low propensity for risk-taking and alcohol consumption. Conclusions: This study not only sheds light on the types of nudge measures that are likely to be more acceptable for promoting healthy behaviors, but also establishes a crucial link between behavioral interventions and healthcare policies. Understanding the nuanced factors influencing the public acceptance of nudges contributes to the discourse on the implementation of behaviorally informed health policies and emphasizes the importance of tailoring interventions to align with public values and preferences.

## 1. Introduction

Public health actions are at the core of contemporary achievements for longer and healthier lives. Among these actions, those directed at addressing the socioeconomic determinants of health and interventions aimed at changing the context of a person’s health decisions are shown to have the greatest impact [1]. Nudges are standardly defined as public or private interventions that steer people in a given direction without depriving them of agency [2]. They have become common in public health initiatives and policymaking as a cost-effective alternative to changing people’s behavior without regulations or financial incentives [2]. Drawing on insights from Kahneman’s dual process theory [3], nudging taps into the automatic, faster, and less conscious cognitive shortcuts enabled by what is known as System 1 thinking as opposed to the reflective, conscious, and logic capacities of System 2 thinking.

Sharing the common denominator of behavioral conditioning techniques [4], the literature proposes different typologies. According to the Nuffield Ladder of Intervention, there are eight ascending intervention levels spanning from “hands off” to “intrusive intervention”. Mongin and Cozic [5] provide an interesting discussion about the types of nudges, concluding that there are mainly two: those which include interventions that interfere minimally in the choice and those which mitigate the negative effects of System 1-based choices. Thaler and Sunstein [6] divide them into: (i) providing information (e.g., labels on cigarette packs); (ii) changes in the physical environment; (iii) changes to standard policy (e.g., the citizen is automatically enrolled in a plan or activity); and (iv) using social norms and salience to generate fictitious information which may influence citizens to choose what is supposedly desired by the majority..

Whilst there is evidence to suggest that nudges can be effective in the short term [7], it is concerning if the deliberate choices of citizens are called into question [8]. This is a debated risk even though not all nudges target behavioral biases (e.g., reminders, warnings, default rules, recommendations, or mandatory disclosure of relevant information) [2,9] and do not replace policy-design, rather improving or complementing it (e.g., [10,11]).

A series of landmark studies [12] found that gain-framed messages worked better in prevention behavior, whereas loss-framed messages encouraged detection behavior. According to the taxonomy proposed by Thaler and Sunstein [6], social norm information also tends to encourage desired health behavior, especially when combined with other techniques [13,14].

Ledderer et al. [15] conducted a systematic literature review of 66 articles (published between January 2008 and April 2019), describing the implementation and efficacy of nudging interventions on health and lifestyles in natural and experimental settings and showing the diversity of the techniques employed. The majority (n = 42) were proven to be effective, 10 reported no effect (e.g., stair climbing, healthy labeling of food, and a multiple-food choice intervention) and the remaining presented mixed results. It is hypothesized that the low impact of nudges that require physical activity may be attributed to the participants’ ability to recognize and reject manipulation. The varying degree of acceptance of these measures across cultural contexts is dependent on the level of intrusiveness of the nudge, its perceived efficacy, and the intention of the choice architect (e.g., scientists nudging hypothetical interventions are more trustworthy than governments pushing forth non-hypothetical ones) [16]. Culturally, as showed by Sunstein et al. [7], most nudging interventions have been conducted in countries with politically liberal climates. Evidence also shows that more lenient interventions are more likely to be accepted, as they cause less disruption to individuals’ daily routines and do not require significant personal changes. In turn, the more a nudge disrupts daily life or requests personal alterations, the less inclined one is to accept it. Less intrusive nudges are typically those that provide additional information (for example, calorie content or health information), as opposed to default-based measures which may trigger psychological reactance and result in contrarian behavior (e.g., [17,18]). As such, individuals who smoke or drink more tend to disagree with the related nudges to decrease their consumption [7], a pattern also found in those with a higher risk aversion.

It is imperative that policymakers take into consideration the potential trade-off between the higher acceptability of less intrusive nudges and their potential effectiveness. As suggested by various studies, including [2,7], combining both types of interventions may be necessary in order to achieve the desired outcomes. Given the disparity in opinions regarding the potential of nudges, it is advisable to subject interventions to a thorough appraisal of their impact.

In this study, we adapt a survey designed to access the attitudes towards health-related public policies based on different types of nudges [7].

In Portugal, there is a growing interest in leveraging behavioral insights to inform the development of health policies. During the COVID-19 pandemic, a ‘task force’ comprised of behavioral scientists was established to advise on the creation of studies aimed at helping the government effectively communicate recommended behaviors to combat the virus. The group’s objective was to contribute to a shift in both individual and collective behaviors, thereby fostering the improvement of public health in Portugal. While some general data on pandemic-related behaviors was collected, the primary focus was on providing policy recommendations and conducting literature reviews [19]. This endeavor in behavioral insights is also being championed by the Competence Centre for Planning, Policy, and Foresight in Public Administration (PlanAPP), a recently established institution dedicated to supporting the definition, implementation, and analysis of public policies.

Despite these efforts, the use of nudges in Portugal is still in its early stages, and there has been no comprehensive analysis of its reception among lay or expert persons and groups in this context. Considering the significance of political sciences and governance in shaping public health policies [18], we specifically target students studying political science and public administration from nine universities located in the north, center, and south of Portugal.

The rationale for this choice is manifold. First, students of public administration and political science are likely to become advocates for or against specific policies in their roles as public servants, politicians, or researchers. By understanding their attitudes towards nudge initiatives, we can identify potential champions who may promote or oppose these policies in the public sphere. In fact, their perceptions can influence public discourse and policy development.

Second, younger generations often have different perspectives on governance and policymaking compared to older generations. Accordingly, analyzing the perceptions of students can shed light on evolving cultural and generational attitudes towards nudging as a governance tool, which in turn can help policymakers adapt their strategies to align with changing societal values.

Third, students may be more receptive to innovative approaches like nudge initiatives, or they simply may exhibit early resistance. Understanding their attitudes can thus provide early indications of potential challenges or opportunities for the implementation of nudge policies in the future.

Based on the available literature [6,7,17,18], we propose the following exploratory hypotheses:

**H1:**  *Nudging approval will be higher for less intrusive nudges;*

**H2:**  *Nudging approval will be higher among participants with a higher degree of trust in institutions;*

**H3:**  *Nudging approval will be lower for participants with higher risk aversion;*

**H4:**  *The nudging approval of lifestyle choices will be lower in participants who smoke or drink.*

## 2. Materials and Methods

### 2.1. Sample

A survey was conducted among students from nine Portuguese universities in which the total amount of vacancies at BSc (3 years) and Master levels are 2608. In total, 240 responses were collected following a purposeful sampling. Although a lack of resources and time limited the process of data gathering, the calculation of the minimum number of participants needed to meet the defined statistical constraints indicates that within a universe of 2608 potential participants, with a sample size of 240 at a confidence level of 95%, we can assume that the real value is within 6.1% of the measured value. The majority (60.8%, n = 146) were female and 37.9% (n = 91) were male.

The majority (95.8%) were Portuguese, mainly from the NUTSII Center (39.6%), Lisbon (35%), and the northern region (19.6%). The participants were mainly young adults, with 57.1% between 20–23 years old, 16.7% between 17-19 years old, 15% between 24–28 years old, and 6.3% between 29–39 years old.

### 2.2. Instruments, Data Collection, and Analysis

The survey included an informed consent form explaining in detail the process, purpose, data anonymity, and participants’ right to withdraw from the study in accordance with the 1975 Declaration of Helsinki and the 2019 Portuguese rules on the General Law for the Protection of Personal Data. It includes a section of items on health, safety, and environmental nudges that were adapted and translated into Portuguese from the original version used in Sunstein et al. [6]. The items were translated back into English by a bilingual speaker. We added one item to the original list of 15, which was presented in a randomized order with two equally phrased dichotomous options, “approve” and “disapprove.” We also adapted a measure of political attitudes, trust in government and institutions, perceived individual health, smoking and drinking habits, a question related to belief in the free market as the best way to solve environmental and economic problems, and socio-demographic variables.

E-mails were sent to student associations and the directors of BSc and MSc courses from the nine universities, who were asked to share the online survey link using Google Forms among their students. A second round of e-mails was needed and, in some cases, personal contacts were pursued to obtain a positive response to disseminate the survey among students. The data were collected between March and May 2022.

The data were analyzed using SPSS version 28.0.1. Descriptive statistics were computed for all variables characterizing the study. To evaluate the psychometric characteristics of the Portuguese version of the survey on the acceptability of nudges, a principal components analysis (PCA) with varimax rotation was employed. Additionally, Kaiser–Meyer–Olkin (KMO) was applied to assess sampling adequacy. Approval rates for the types of nudges identified through PCA were then examined using descriptive statistics (mean (M) and standard deviation (SD)). Bivariate correlations were used to investigate the relationship between the approval rates of nudges and trust in institutions and groups. Finally, two independent sample t-tests were conducted to compare nudge agreement in groups of participants with and without a history of alcohol and cigarette consumption.

## 3. Results

### 3.1. Descriptive Characterization

Descriptive statistics were conducted on the variables of characterization of the sample (Table 1). Most respondents feel that they are healthy (M = 5.25, SD = 1.1). 81.3% do not smoke, and 69.2% drink alcohol once or twice a week. While a relatively substantial number of respondents reported being willing to take chances (M = 5.74; SD = 0.97), at the same time they perceive a low level of agency, choice, and control over their decisions (M = 3.41, SD = 1.66). The concern for the environment was relatively high (M = 4.49; SD = 1.5), as was the level of trust in the free market to solve economic and environmental issues (M = 4.54, SD = 1.5). The participants reported trusting the United Nations (M = 4.68; SD = 1.43), the army (M = 4.58; SD = 1.57), and the police (M = 4.58; SD = 1.57) the most. Public Administration (M = 3.41, SD = 1.41) and Political Parties (M = 3.77, SD = 1.4) were the least trusted. The highest level of trust was reported for people they knew personally (M = 5.85; SD = 1.46).

### 3.2. Nudge Approval: Overall and by Type

Of the respondents, 48.8% approved six to ten nudges and 42.9% approved 10 to 16 nudges. Only 8.3% agreed with less than five items. This suggests that several health-related nudges are favorably regarded to be applicable in real-life settings.

To assess the psychometric characteristics of the Portuguese version, a principal component analysis (PCA) with varimax rotation was conducted on the list of 16 nudges. Three factors were retained with eigenvalues greater than one. All items had loadings above 0.40 and were kept. The Kaiser–Meyer–Olkin (KMO) measure of sampling adequacy indicated that the sample was adequate for the adjustment of the data (t (240) = 0.796; *p* < 0.001), and Bartlett’s χ^2^ was equal to 368.9 with *p* < 0.000.

The dimensions identified (Table 2) align with two of the levels proposed by Thaler and Sunstein [6]. The first dimension (referred to as Type 1) encompasses nudges associated with providing on-site nutrition information (α = 0.671). The second dimension (Type 2) includes both changes in the physical environment and modifications to standard policy nudges (α = 0.869). Type 3 incorporates nudges using social norms and salience through public education (α = 0.567). Type 1 and Type 3 are characterized as less intrusive types of nudges, focusing on information provision and leveraging social norms. In contrast, Type 2 includes changes to the physical environment and related policies, making it a more comprehensive and impactful category.

An analysis of the approval rates by the types extracted in the PCA (Table 3) showed that acceptance of the less intrusive nudges, particularly from Type 1 (M = 0.78; SD = 0.26) and Type 3 (M = 0.75; SD = 0.27) is higher. For example, the nudge with the highest approval (95%) refers to a public awareness campaign to fight child obesity, based on providing information, followed by the inclusion of calorie tables in fast food chain restaurants (88.3%).

The lowest rates of approval are for more intrusive measures of all types, but particularly of those of Type 2 (M = 0.5; SD = 0.26) taken by the government, either through including subliminal messages to discourage people from smoking and eating too much (33.3%), imposing default contributions on airlines to help the environment (33.3%), or on taxes that revert to the Red Cross (41.2%).

The measures that received greater approval tend to be framed as either positive or neutral. For example, calorie tables in fast food chain restaurants, federal government-mandated labels on products with unusually high salt levels, prominent placement of the healthiest food in large grocery stores, and a national government campaign to reduce child obesity by providing information to parents for making healthier choices for their children.

Accordingly, it is a common trend among the three types of nudges to have the lowest approval rate when they intervene excessively in the architecture of choice by limiting options, provoking unpleasant emotions related to unhealthy lifestyle choices, or imposing a default contribution that requires an additional step to opt out.

Bivariate correlations were conducted between the approval rates and trust in institutions and groups. No statistically significant correlation was found between approval and trust in institutions (r (240) = 0.057, *p* < 0.38), but approval significantly correlates with trust in groups (r (240) = 0.141, *p* < 0.5).

Two independent sample t-tests were conducted between participants who consume and do not consume alcohol and cigarettes. The participants who drink alcohol tend to agree less with nudges, and the differences are statistically significant (t (240) = 2.68, *p* = 0.08). No significant differences in nudge approval were found between those who smoked and those who did not (t(240) = 2.38, *p* = 0.16).

A lower propensity to take risks predicts a higher approval (β = 0.23; 185) (F (240) = 7.4; *p* < 0.001).

## 4. Discussion

This study aimed to evaluate the level of endorsement of nudges that promote health policies and their underlying factors among Portuguese students enrolled in governance and health policy-related academic programs.

The approval rate of less intrusive nudges is higher, which evokes the psychological reactance found when more intrusive default-based nudges were applied (e.g., [17,18]).

Our H1, posing that less intrusive measures would be more accepted, was thus confirmed, corroborating previous evidence. Notwithstanding, and particularly in health-related behaviors, these less intrusive measures, such as providing information strategically, are considered less efficient [6]. From the viewpoint of health policy making, should less intrusive, but also potentially less efficient measures, be more adopted because citizens tend to agree more with them? Or should the expected efficacy be a more relevant criteria, regardless of the manipulative nature of the intervention? Being nudges mediated by ‘System 1heuristics, if there is a well-known disapproval of more intrusive measures (hence relying more on System 1), their application taps directly into the concerns of the critics about preserving citizens’ deliberate choices [14].

In our sample, the results partially mirrored the typology proposed by Sustein [6] with only three types: nudges providing information; a combination of nudges that advocate for changes in the physical environment and changes to standard policy; and a third one drawing on social norms and salience through public education.

It is worth noting that our design did not permit the assessment of framing effects, and as such, we cannot draw inferences about the impact of content and framing on responses. However, our findings are consistent with existing literature, which suggests that gain-framed messages play a moderating role in the effectiveness of health messages, particularly in the context of preventive behaviors [12].

H2 was partially confirmed with the positive association between a higher level of trust within groups and a higher level of approval. Notably, the degree of trust in political parties and parliament was generally low in our sample, and this is compounded by the skepticism often faced by government-based interventions. This skepticism may also be applicable in this context. Since the degree of trust in government-based strategies tends to be lower, the initiatives will more likely succeed if someone independent (e.g., a group), preferably recognized as knowledgeable and with no agenda, is involved in the process [16].

The moderate-to-high degree of approval suggests that our participants’ attitudes do not echo the critics’ argument of compromising citizens’ actual deliberate choices.

Consistent with the findings in [7], risk aversion predicts nudge approval as a measure, thereby confirming H3. H4 received partial confirmation. Similar to the results in [7], no significant differences were observed between smokers and non-smokers. However, differences did emerge between individuals who consume alcohol and those who do not.

## 5. Conclusions

This study offers insights into some key variables that may influence nudge acceptance, namely the level of disruption and framing. Despite the surveyed sample not being representative of the general Portuguese population, it may be understood as a proxy of an expert sample, considering their anchoring on political science and public administration. Tackling students in similar studies can serve an educational purpose: on the one hand, it can help raise awareness among future decision-makers about the concept of nudging and its implications; on the other hand, it can encourage academic institutions to open the debate on the perks and shortcomings of behavioral economics and nudge theory into their curricula, hence better preparing students for future roles in governance.

The study has several limitations, with the primary one being the sample size and composition, which is not representative of the Portuguese context. Nevertheless, our analysis of these patterns in a non-lay sample of students majoring in political science and public administration suggests a potential inclination among those who may become key stakeholders in related decision-making. Future research steps should aim to, on the one hand, include students of health-related areas (e.g., public health or health management) and, on the other hand, to survey a representative sample of the Portuguese population. The research would also benefit from including qualitative measures, such as a free-evocation task or open-ended questions, to gain insights into how participants perceive and represent this type of strategy. This additional information would enhance data interpretation.

Furthermore, comprehensive and practical information is essential for health policymakers if they intend to incorporate this strategy into their repertoire.

## Figures and Tables

**Table 1:** Means and Standard Deviations of Trustworthiness in Institutions and Groups.

Trustworthiness	M	SD
**Institutions**		
United Nations	4.68	1.432
Army	4.58	1.572
Police	4.25	1.545
European Union	4.20	1.419
University	3.97	1.517
Parliament	3.81	1.515
Political parties	3.77	1.424
Public Administration	3.41	1.417
**Groups**		
People you know personally	5.85	1.466
People with another religion	4.93	1.426
Your family	4.55	1.568
People you know for the first time	4.25	1.611
Your neighborhood	4.19	1.668
People with another nationality	2.94	1.324

**Table 2:** Principal Component Analysis.

	Type 1	Type 2	Type 3
**Providing information on nutrition**			
Calorie tables at fast food chain restaurants.	0.827		
Traffic light system for food (healthy foods with a small green label, unhealthy foods with a small red label, and foods that are neither especially healthy nor especially unhealthy with a small yellow label).	0.704		
The federal government requires labels on products that have unusually high levels of salt, as in “This product has been found to contain unusually high levels of salt, which may be harmful to your health.”	0.536		
For reasons of public health and climate protection, the government requires canteens in public institutions (schools, public administrations, and similar) to have one meat-free day per week.	0.525		
**Healthier lifestyles and changes to standard policy**			
To foster a practice of physical exercise, your institution by default subscribes to gym classes with a symbolic value. You could revoke the subscription if you want.		0.796	
A university enrolls you in college formal groups or associations. If the student participates they are awarded 3 credits. If opting out, a request must be made.		0.734	
The government requires airlines to charge people, with their airline tickets, a specific amount to offset their carbon emissions (about €10 per ticket); under the program, people can opt out of the payment if they explicitly say that they do not want to pay.		0.713	
The government assumes, on tax returns, that people want to donate 50 euros to the Red Cross (or another charity), subject to opting out if people explicitly say that they do not want to make the donation.		0.712	
The government requires movie theaters to include subliminal messages to discourage people from smoking and eating too much.		0.708	
Award points to the final grade in the case of students attending 100% of their classes.		0.587	
To halt the child obesity problem, the government requires large supermarket chains to keep cashiers free of sweets.		0.526	
Large grocery stores to place their most healthy foods in a prominent, visible location.		0.437	
The government requires large electricity providers to adopt a system in which consumers are enrolled in a green energy supplier, but may opt out if they wished.		0.312	
**Using social norms and salience through public education**			
To reduce deaths and injuries associated with distracted driving, the national government adopts a public education campaign with vivid and graphic stories and images, designed to discourage people from texting, emailing, or talking on their cellphones while driving.			0.804
To reduce child obesity, the national government adopts a public education campaign, providing information to parents to choose healthier options for their kids.			0.652
The government displays public awareness messages to discourage people from smoking and drinking.			0.561

**Table 3:** Nudge approval by type.

	Approval (N)	Approval (%)
Type 1: Calorie tables at fast food chain restaurants.	212	88.3%
Type 1: The federal government requires labels on products that have unusually high levels of salt, as in “This product has been found to contain unusually high levels of salt, which may be harmful to your health”.	201	83.8%
Type 1: Traffic light system for food (healthy foods with a small green label, unhealthy foods with a small red label, and food that are neither especially healthy nor especially unhealthy with a small yellow label).	188	78.3%
Type 1: For reasons of public health and climate protection, the government requires canteens in public institutions (schools, public administrations, and similar) to have one meat-free day per week.	155	64.6%
Type 2: The government requires large electricity providers to adopt a system in which consumers are enrolled in a green energy supplier, but may opt out if they wish.	184	76.7%
Type 2: The government requires for movie theaters to include subliminal messages to discourage people to smoke and eat too much.	177	73.8%
Type 2: Award points to the final grade in the case of students attending 100% of their classes.	140	58.3%
Type 2: To halt the child obesity problem, the government requires large supermarket chains to keep cashiers free of sweets.	126	52.5%
Type 2: A university enrolls you in college formal groups or associations. If the students participate, they are awarded 3 credits. If opting out, a request must be made.	123	51.2%
Type 2: To foster a practice of physical exercise, your institution by default subscribes to gym classes with a symbolic value, you could revoke the inscription if you want.	110	45.8%
Type 2: The government assumes, on tax returns, that people want to donate 50 euros to the Red Cross (or another charity), subject to opting out if people explicitly say that they do not want to make the donation.	96	41.2%
Type 2: The government requires airlines to charge people, with their airline tickets, a specific amount to offset their carbon emissions (about €10 per ticket); under the program, people can opt out of the payment if they explicitly say that they do not want to pay.	82	34.2%
Type 2: The government requires movie theaters to include subliminal messages to discourage people from smoking and eating too much.	80	33.3%
Type 3: To reduce deaths and injuries associated with distracted driving, the national government adopts a public education campaign with vivid and graphic stories and images, designed to discourage people from texting, emailing, or talking on their cellphones while driving.	188	78.3%
Type 3: To reduce child obesity, national government adopts a public education campaign, providing information to parents to choose healthier options for their kids.	228	95%
Type 3: The government displays public awareness messages to discourage people from smoking and drinking.	123	51.2%

## Data Availability

The data used to support the findings of this study will be available from the corresponding author upon reasonable request.
